# ^18^F-FDG PET/CT-derived parameters for differentiating colorectal carcinoma from adenoma

**DOI:** 10.3389/fonc.2026.1693100

**Published:** 2026-02-18

**Authors:** Ting Li, Junhu Wang, Yun Zhang

**Affiliations:** 1 Department of Nuclear Medicine, Yuncheng Central Hospital Affiliated to Shanxi Medical University, Yun Cheng, China; 2 Department of Critical Care Medicine, Yuncheng Central Hospital Affiliated to Shanxi Medical University, Yun Cheng, China

**Keywords:** 18F-FDG, adenomas, colorectal carcinomas, parameters, PET/CT

## Abstract

**Objective:**

The aim of this study is to evaluate the ability of ^18^F-FDG PET/CT-derived parameters to differentiate between colorectal cancer and adenoma.

**Methods:**

One-hundred and twenty-five patients with colorectal cancers and 57 with colorectal adenomas were retrospectively analyzed. Parameters assessed on ^18^F-FDG PET/CT included maximum standardized uptake value (SUVmax), metabolic tumor volume (MTV), and total lesion glycolysis (TLG). Mann–Whitney U-test was used to compare parameters between groups, and receiver-operating characteristic (ROC) curve analysis was performed to assess their discriminatory performance.

**Results:**

Colorectal carcinomas demonstrated significantly higher SUVmax (P = 0.003), SUVmean (P = 0.005), MTV (P < 0.001) and TLG (P< 0.001). The AUCs for differentiating colorectal carcinomas and adenomas were 0.657, 0.649, 0.979, and 0.987, for SUVmax, SUVmean, MTV, and TLG, respectively. The optimal cut-off value for MTV and TLG was 3.5 and 22.6, respectively, yielding a high sensitivity and specificity (96.5% and 92.5% for MTV, 96.5% and 95.5% for TLG). The optimal cut-off value for SUVmax and SUVmean was 8.8 and 4.4, respectively, yielding a sensitivity of 88.6% for SUVmax and 95.6% for SUVmean, and a low specificity of 42.5% for SUVmax, and 32.5% for SUVmean.

**Conclusions:**

Of the ^18^F-FDG PET/CT parameters investigated, MTV and TLG showed high accuracy in distinguishing colorectal carcinoma from adenomas, and were significantly higher in colorectal carcinomas than in adenomas.

## Introduction

^18^F-FDG PET/CT has widespread clinical application in the diagnosis and management of both malignant and benign disease. Today, ^18^F-FDG PET/CT has established roles in the staging, restaging and follow-up of colorectal carcinomas. However, ^18^F-FDG PET/CT may result in incidental uptake across a wide range of organs, including the colon and rectum. Incidental colorectal uptake has been reported in about 1.3% to 2.7% of patients who underwent ^18^F-FDG PET/CT (1, 2). Diffuse and longitudinal uptake in the colon and rectum are commonly due to physiologic or inflammatory process (3–5), but focal colorectal uptake may be indicative of either benign or malignant disease. When incidental focal colorectal FDG uptake is detected, a colonoscopy is usually recommended for diagnostic clarification or to rule out malignant disease.

Colorectal adenomas are common lesions with a prevalence of approximately 15.6%, and the most common type is tubular adenoma (6). The incidence of colorectal adenomas increases with age (7), and their progression to colorectal carcinoma follows the adenoma–carcinoma sequence, which is influenced by multiple factors including histological subtype (e.g., villous adenomas have higher malignant potential than tubular adenomas), lesion size (adenomas >10 mm are associated with increased risk), and grade of dysplasia (high-grade dysplasia confers higher malignancy risk) (8, 9). Previous studies have reported that the cumulative risk of progression to carcinoma ranges from 1% to 10% over 10 years, rather than a fixed 5% (10, 11). They can cause medical dilemma due to intense uptake on ^18^F-FDG PET/CT (12–14). Although colonoscopy is recommended in these cases, a noninvasive imaging method for identifying malignant lesions is crucial. Maximum standardized uptake value (SUVmax) is a popularly used metabolic parameter to help distinguish between benign and malignant disease. However, the ability of SUVmax in differentiating colorectal carcinomas from adenomas still remains limited (15, 16)). Few studies to date, however, have evaluated the use of other metabolic parameters, including metabolic tumor volume (MTV), and total lesion glycolysis (TLG), in the differentiate between colorectal carcinomas and adenomas. The aim of this study is to evaluate the ability of ^18^F-FDG PET/CT-derived parameters, including SUVmax, SUVmean, MTV, and TLG, to differentiate between colorectal carcinoma and adenoma. We hypothesize that volumetric metabolic parameters (MTV and TLG), which integrate both metabolic activity and lesion volume, will demonstrate superior diagnostic accuracy compared to SUV-based metrics (SUVmax and SUVmean) in differentiating colorectal carcinoma from adenoma.

## Methods

### Patients

The medical records of patients with pathologically confirmed colorectal carcinomas and patients with pathologically confirmed colorectal adenomas who underwent ^18^F-FDG PET/CT between January 2022 and January 2025 were retrospectively reviewed. The study was approved by the Institutional Review Board of our hospital, and the requirement of informed consent was waived due to the retrospective nature of the study.

All included patients underwent ^18^F-FDG PET/CT for the following clinical indications: 68 patients for suspected colorectal disease (e.g., positive fecal occult blood test, abdominal pain, or altered bowel habits), 47 patients for colorectal cancer staging, and 67 patients for oncological follow-up of other malignancies (e.g., lung cancer, breast cancer) with incidental focal colorectal FDG uptake. Focal colorectal uptake was defined as a discrete, localized area of increased FDG activity that was distinguishable from surrounding normal colonic mucosa and not consistent with physiological (e.g., uniform uptake in the cecum due to bacterial activity) or inflammatory (e.g., diffuse, segmental uptake in ulcerative colitis) uptake patterns. Patients with FDG-negative colorectal lesions were excluded because the study aimed to evaluate the discriminatory ability of metabolic parameters in lesions with detectable FDG uptake, which is the clinical scenario where differentiation between carcinoma and adenoma is most challenging. In our clinical practice, patients with suspected colorectal lesions or incidental focal uptake on PET/CT are routinely referred for colonoscopy regardless of the primary indication for PET/CT.

### ^18^F-FDG PET/CT acquisition and image interpretation

All the included patients fasted for at least 6 h before ^18^F-FDG PET/CT with blood glucose level of less than 200 mg/dL. ^18^F-FDG was intravenously injected at a dose of 5.55 MBq/kg and PET/CT images were acquired 1 h later. Low-dose CT images were initially acquired from the skull base to the upper thigh for PET attenuation correction and anatomical reference using the following parameters: a tube voltage of 120 kV, tube current of 150 mA, 3.75 mm of slice thickness, 1.375 of pitch, and 0.8 s of rotation speed. PET was then performed in the same bed position in 3D mode (2 min/bed position). All scans were performed with dedicated PET/CT scanners (Discovery MI, GE Healthcare, Milwaukee, WI, USA). The VPFX-S algorithm (2 iterations, 24 subsets, 4 mm Gaussian post filter) was used for image reconstruction.

All PET/CT images were reviewed by 2 experienced nuclear medicine physicians (with 8 and 12 years of experience in PET/CT interpretation) who were blinded to the pathological results. Qualitative visual assessment was performed to classify lesions as hypermetabolic, defined as FDG uptake greater than that of the surrounding normal colonic mucosa. Discrepancies between the two physicians were resolved by consensus. A region of interest (ROI) was drawn around the colorectal lesion while avoiding the peripheral area. All values of SUVmax, MTV, TLG were measured by the analysis software (Medex-NM imaging analysis system) for each lesion.

### Statistical analysis

Patient data and quantitative parameters on ^18^F-FDG PET/CT were expressed as mean ± SD. Quantitative parameters were compared using Mann–Whitney U-test. The cut-off value of parameters to differentiate colorectal carcinoma from adenoma was evaluated by receiver operating characteristic (ROC) curve analysis. The areas under the curve (AUC) and the sensitivity and specificity of differential diagnoses were also calculated. For all tests, *P* value<0.05 was considered statistically significant. All statistical analyses were performed using SPSS Statistics for Windows version 21.0 (IBM Corp, Armonk, NY, USA).

## Results

### Patient characteristics

A total of 182 patients were finally included in this study, including 125 patients with colorectal carcinoma and 57 patients with colorectal adenoma. Of the 125 patients with colorectal carcinomas, 70 were female and 55 were male with median age of 65 (IQR: 56–71) years. Of the 57 patients with colorectal adenomas, 34 were female and 23 were male with median age of 64 (IQR: 59–70) years. Patient characteristics were shown in **Table 1**.

**Table 1:** Demographic and clinical characteristics of the patients included in this study.

Characteristic	Colorectal carcinomas (n=125)	Colorectal adenomas (n=57)	Total (n=182)
Gender (F:M)	70:55	34:23	104:78
Age, years (Median, IQR)	65 (56–71)	64 (59–70)	65 (58–71)
Location of colorectal lesions, n (%)			
Ascending Colon	27 (21.6)	11 (19.3)	38 (20.9)
Transverse Colon	7 (5.6)	9 (15.8)	16 (8.8)
Descending Colon	15 (12.0)	8 (14.0)	23 (12.6)
Sigmoid Colon	38 (30.4)	19 (33.3)	57 (31.3)
Rectum	38 (30.4)	10 (17.5)	48 (26.4)

### PET/CT performance

**Figure 1:**
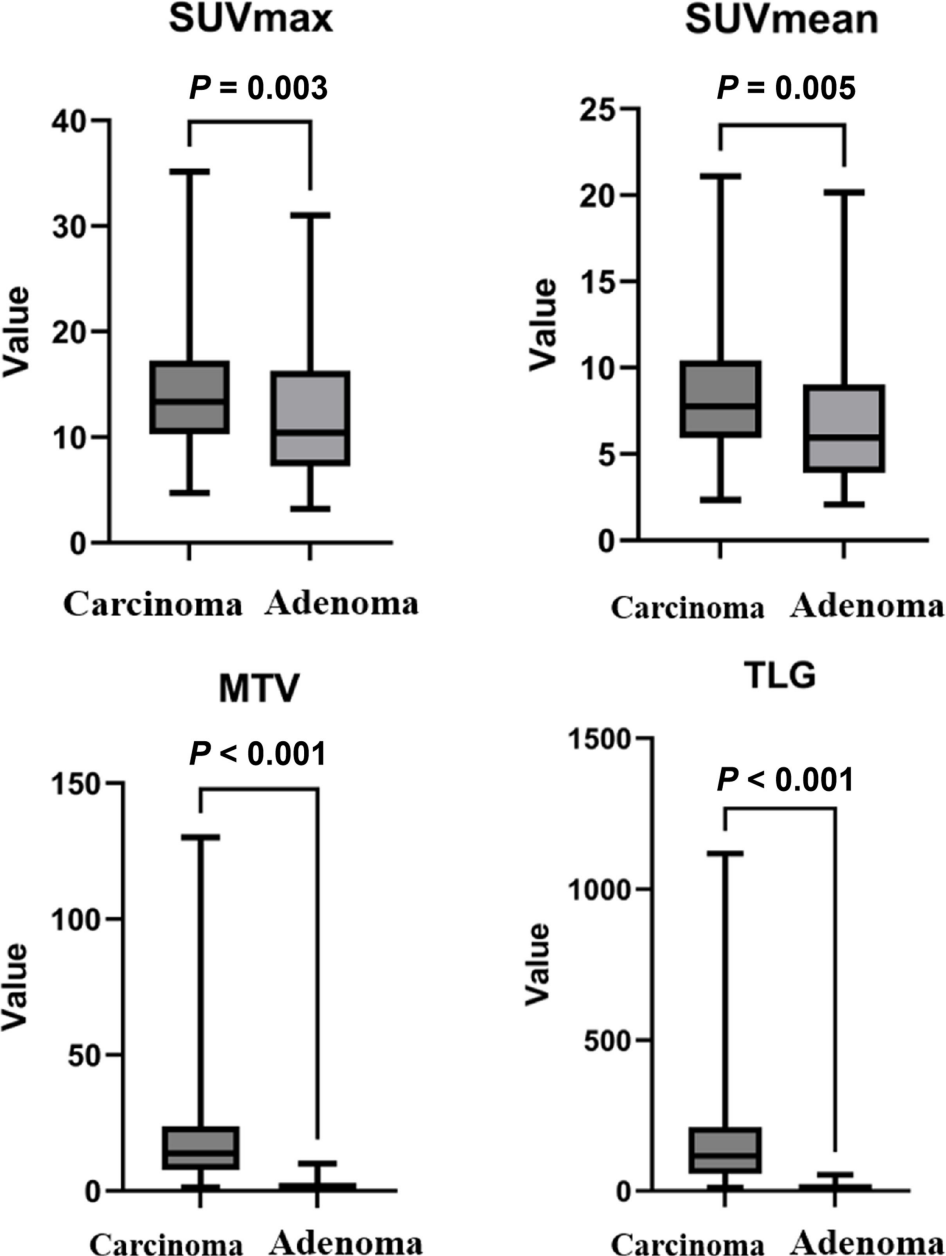
Comparison of ^18^F-FDG PET/CT-derived metabolic parameters in patients with colorectal carcinoma and adenomas.

All patients with colorectal carcinomas and adenomas demonstrated high uptake on ^18^F-FDG PET/CT. For the 125 patients with colorectal carcinomas, the median SUVmax was 13.4 (IQR: 10.3–17.3), SUVmean was 7.7 (IQR: 5.9–10.3), MTV was 13.7 (IQR: 7.7–23.5), and TLG was 114.9 (IQR: 56.6–210.2). For the 57 patients with colorectal adenomas, the median SUVmax was 10.4 (IQR: 7.2–15.5), SUVmean 5.9 (IQR: 3.9–8.8), MTV 0.9 (IQR: 0.6–1.3), and TLG 6.0 (IQR: 3.3–10.0). The investigated quantitative parameters SUVmax (*P* = 0.003), SUVmean (*P* = 0.005), MTV (*P* < 0.001), TLG (*P* < 0.001) were all significantly higher in colorectal carcinomas than in adenoma (**Figure 1**; **Table 2**).

**Table 2:** Comparison of metabolic parameters of carcinomas and adenomas.

Metabolic parameters	Carcinomas (median, IQR)	Adenomas (median, IQR)	P value
SUVmax	13.4 (10.3–17.3)	10.4 (7.2–15.5)	0.003
SUVmean	7.7 (5.9–10.3)	5.9 (3.9–8.8)	0.005
MTV	13.7 (7.7–23.5)	0.9 (0.6–1.3)	<0.001
TLG	114.9 (56.6–210.2)	6.0 (3.3–10.0)	<0.001

### ROC analysis of metabolic parameters

**Figure 2:**
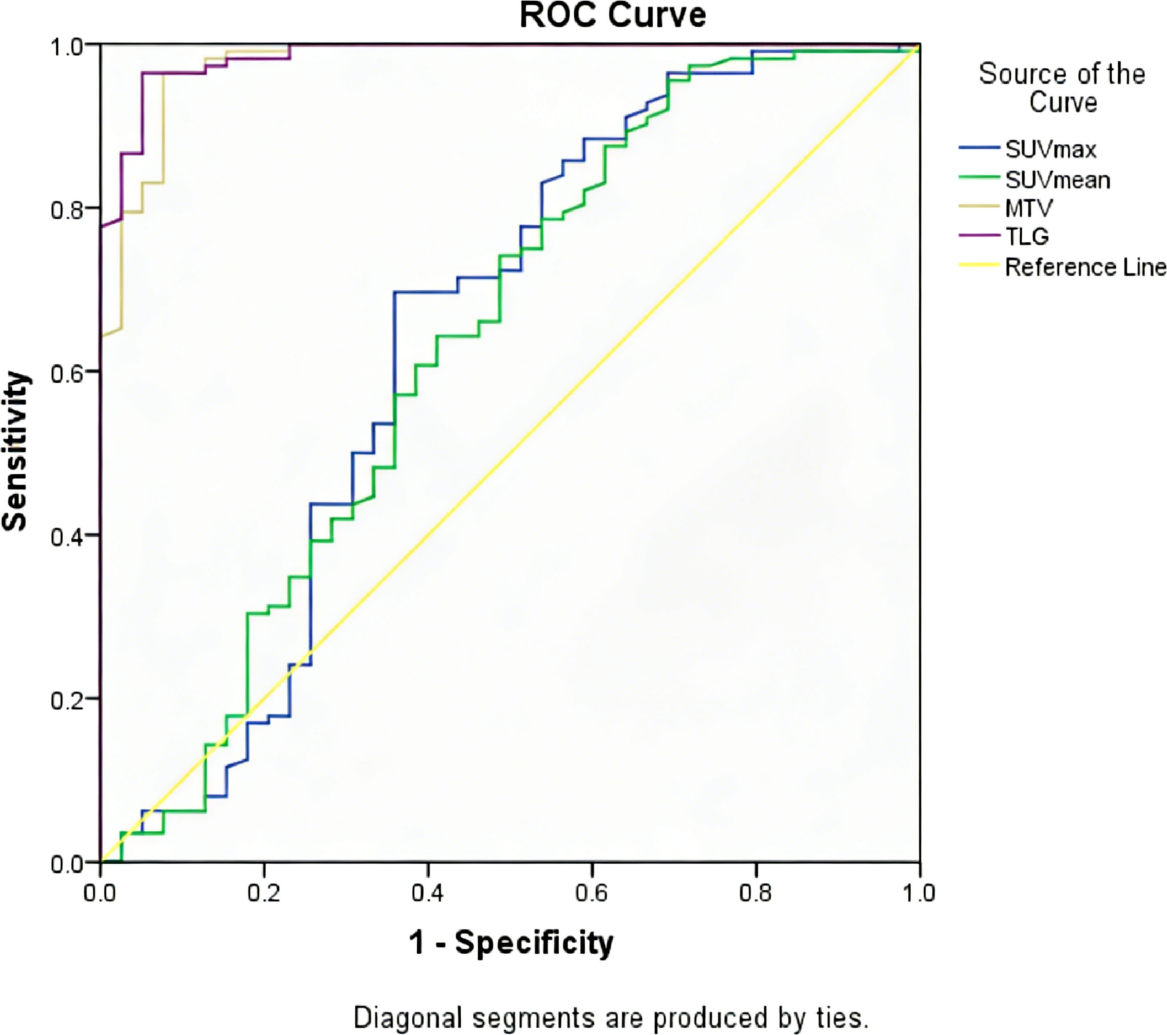
Comparison of area under receiver-operating characteristics curve among metabolic parameters in differentiating colorectal carcinomas from adenomas.

**Figure 3:**
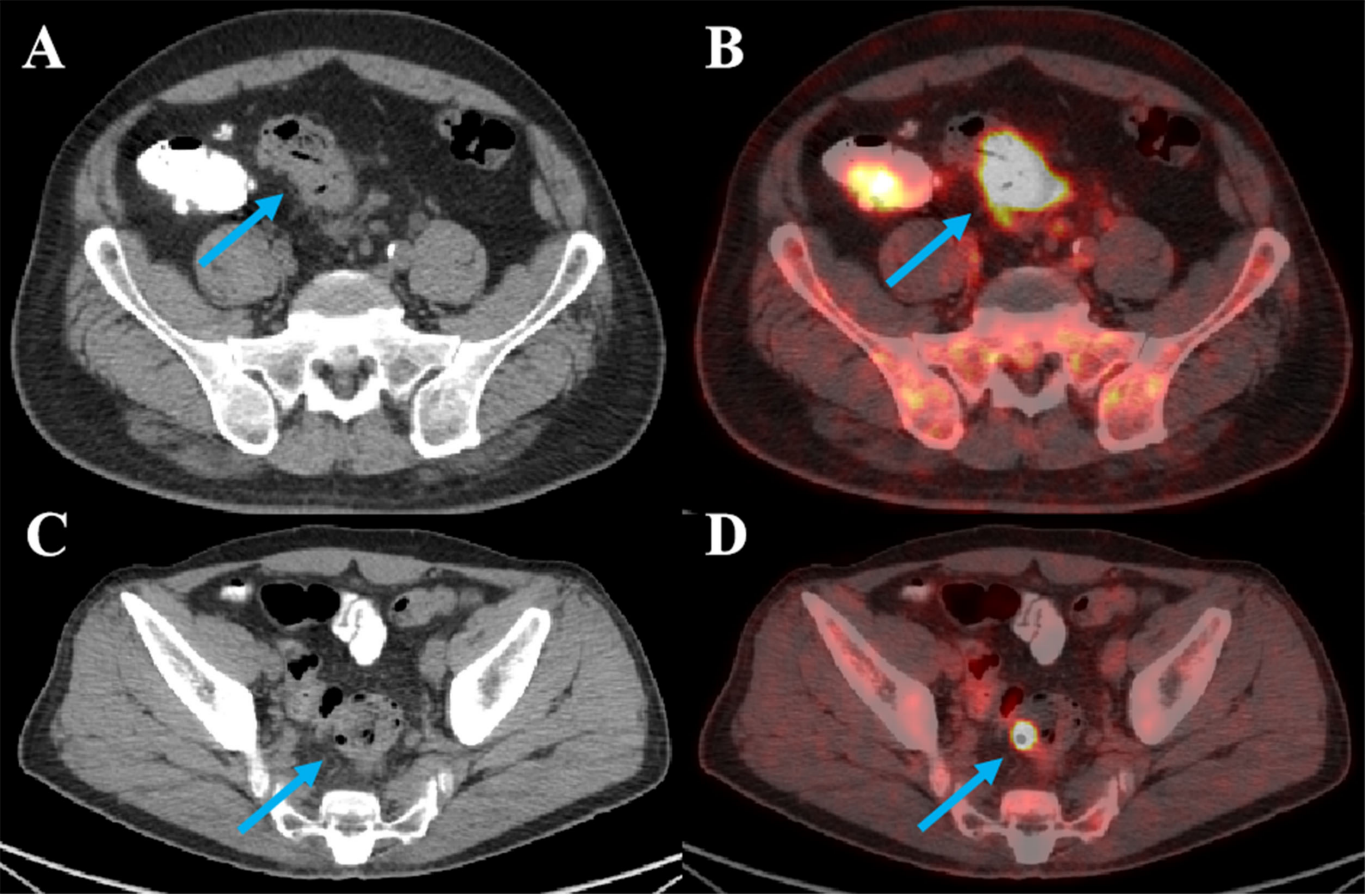
Representative ^18^F-FDG PET/CT images of colorectal carcinoma and adenoma. **(A, B)** A 51-year-old male patient with carcinoma of sigmoid colon. The tumor showed strong FDG uptake with SUVmax of 16.6, SUVmean of 8.3, MTV of 12.7, and TLG of 105.4. **(C, D)** A 61-year-old male patient with tubulovillous adenoma of sigmoid colon. The tumor also showed strong FDG uptake with SUVmax of 25.6, SUVmean of 15.3, MTV of 0.3, and TLG of 4.0.

The sensitivity and specificity of SUVmax, SUVmean, MTV, and TLG were summarized in **Table 3**. The ROC curve analysis showed that TLG and MTV had the highest diagnostic accuracy for distinguishing colorectal carcinoma from adenoma on the basis of AUC (**Figure 2**). TLG displayed the highest AUC value of 0.987 (*P* < 0.001; 95%CI: 0.973∼1.000) among metabolic parameters with a sensitivity of 96.5% and a specificity of 95.0% at an optimal cut-off of 22.6. The AUC value for MTV was 0.979 (*P* < 0.001; 95%CI: 0.957∼1.000) at an optimal cut-off of 3.5 with 96.5% sensitivity and 92.5% specificity. The AUC value of SUVmax and SUVmean was 0.657 (*P* = 0.003), and 0.649 (*P* = 0.005), respectively, with a sensitivity of 88.6% for SUVmax and 95.6% for SUVmean, and a specificity of 42.5% for SUVmax and 32.5% for SUVmean, at an optimal cut-off of 8.8 and 4.4. Representative images of colorectal carcinoma and adenoma are illustrated in **Figure 3**.

**Table 3:** ROC curve analysis of metabolic parameters in differentiating carcinomas and adenomas.

Metabolic parameters	Cut-off value	AUC (95%CI)	Sensitivity (%)	Specificity (%)	P value
SUVmax	8.8	0.657 (0.544∼0.771)	88.6	42.5	0.003
SUVmean	4.4	0.649 (0.539∼0.759)	95.6	32.5	0.005
MTV	3.5	0.979 (0.957∼1.000)	96.5	92.5	<0.001
TLG	22.6	0.987 (0.973∼1.000)	96.5	95.0	<0.001

## Discussion

The present study investigates the use of ^18^F-FDG PET/CT to identify distinctive features distinguishing colorectal carcinoma from adenomas. The quantitative metabolic parameters displayed the potential to differentiate between colorectal carcinoma and adenomas. Among these parameters, MTV and TLG were found to show the highest AUC, exhibiting better diagnostic accuracy than SUVmax and SUVmean.

The characterization of focal FDG uptake is of utmost difficulty in clinical routine because colorectal adenoma may exhibit intense uptake mimicking malignancy. SUVmax is the most commonly used metabolic parameter in clinical practice. To differentiate between colorectal carcinoma and adenoma, several studies have proposed to evaluate the intensity of the FDG uptake, denoted as SUVmax. However, their results were found to be contradictory. Some studies found no significant differences in average SUVmax between colorectal malignant and benign lesions (2, 16–21). Some studies have shown significant difference in average SUVmax between malignant and benign lesions (3, 12, 22–24). Similarly, our results showed that the FDG uptake of colorectal carcinoma, denoted by SUVmax and SUVmean, was significantly higher than adenoma. Luboldt et al. proposed a cut-off value of 5 for SUVmax for distinguishing carcinomas from adenomas (12). In the current study, the optimal cut-off value of SUVmax was found to be 8.8 to differentiate carcinomas from adenomas with a sensitivity of 88.6% and a specificity of 42.5%. Despite the statistically significant difference in average SUVmax, the ability of SUVmax to differentiate carcinomas and adenomas showed be considered with caution given its relatively low specificity (42.5%) and the fact that there was a relatively large overlap in SUVmax values between carcinomas and adenomas. In our study, many of the adenomas had an SUVmax value of higher than 8.8, with the highest SUVmax value of up to 31.0. Therefore, SUVmax might not be recommended as a reliable metabolic parameter for distinguishing carcinoma from adenomas.

MTV is a different parameter measurement, representing the extent of FDG uptake by tumor tissues, and is used to estimate the gross tumor volume. The present study showed that MTV had high diagnostic accuracy in distinguishing colorectal carcinomas from adenomas. A cut-off value of 3.5 rendered a high sensitivity of 96.5% and specificity of 92.5%. This finding was in line with a previous study by Oh et al. who reported that MTV was able to differentiate dysplasia from malignancy with a cut-off value of 3.14cm^3^ and AUC of 0.947 (24). In addition, the difference in MTV between carcinoma and adenoma was more prominent than the difference in SUVmax, and the AUC of MTV was higher than that of SUVmax (0.979 *vs.* 0.657). These findings were also in accordance with the findings of their study. For colorectal adenomas, the malignant potential and increasing dysplasia are known to be associated with the increase in tumor size (10, 11), and the likelihood of carcinoma was also increased with the increase in tumor size (25). These results further supported the ability of MTV in differentiating colorectal carcinomas and adenomas. In this study, we also investigated the value of TLG to differentiate colorectal carcinoma and adenomas. Among the parameters evaluated, TLG displayed the highest AUC, with a cut-off value of 22.6 yielding both high sensitivity and specificity in differentiating the two disease entities. To our knowledge, this is the first study demonstrating the great value of TLG in the differentiation of colorectal carcinoma and adenoma.

**Figure 3** illustrates a notable observation: the adenoma case exhibits a higher SUVmax (25.6) than the carcinoma case (16.6), yet the carcinoma has substantially larger MTV and TLG. This finding supports the notion that lesion size, which is the basis for volumetric parameters (MTV and TLG), may be a more robust discriminator than metabolic intensity alone (reflected by SUVmax and SUVmean) in distinguishing colorectal carcinoma from adenoma. Colorectal carcinomas typically exhibit greater tumor burden, leading to larger MTV, whereas adenomas are generally smaller even when metabolically active. However, it is important to note that TLG integrates both volume and metabolic intensity (TLG = MTV × SUVmean), making it a more comprehensive parameter than either MTV or SUV alone. This integration likely contributes to its highest AUC in our study. Future studies could further explore the relative contributions of lesion size and intrinsic metabolic activity by adjusting for volume in SUV analyses, or by evaluating smaller carcinomas and larger adenomas to minimize size-related confounding.

This study is not without limitation. First, due to the retrospective nature of the study, there was a possibility of selection bias in that the patients who had a high likelihood to receive further colonoscopy were included. Second, colorectal lesions with negative FDG uptake were not included in this study. The clinical utility of ^18^F-FDG PET/CT in terms of the detection rate of colorectal lesions was fully investigated in previous studies (3, 12–14). The current study focused on the characterization of ^18^F-FDG PET/CT-derived metabolic parameters of colorectal carcinoma and adenoma, and the ability of metabolic parameters in the distinguishment of the two diseases.

## Conclusion

Among the ^18^F-FDG PET/CT-derived parameters investigated, MTV and TLG demonstrated high accuracy in distinguishing colorectal carcinoma from adenomas, with optimal cut-off values of 3.5 and 22.6, respectively, yielding excellent sensitivity and specificity. These volumetric parameters were significantly higher in colorectal carcinomas than in adenomas, likely reflecting the greater tumor burden of malignant lesions. Clinically, the use of MTV and TLG may help refine the interpretation of focal colorectal FDG uptake on PET/CT, particularly in patients with incidental uptake or suspected colorectal lesions, and could assist in prioritizing colonoscopy referrals or guiding clinical decision-making. However, this study has limitations: it is a single-center retrospective study with potential selection bias, as only patients with FDG-avid lesions were included; the influence of lesion size on volumetric parameters cannot be fully disentangled from intrinsic metabolic activity; and specific scanner models may affect parameter reproducibility. Future prospective multicenter studies with larger sample sizes and standardized acquisition protocols are warranted to validate these findings and explore the utility of combining volumetric parameters with other imaging or clinical factors.

## Data Availability

The raw data supporting the conclusions of this article will be made available by the authors, without undue reservation.
